# Internet Research: Implications for The Future of Health Care

**DOI:** 10.2196/jmir.1.suppl1.e4

**Published:** 1999-09-19

**Authors:** Ted Shortliffe

**Affiliations:** ^1^ Professor for Medical Informatics, Stanford UniversityUSA

## Abstract

The phenomenal growth in Internet usage, largely due to the success of the World Wide Web, has stressed the international networking infrastructure in ways that were never contemplated when the early ARPAnet emerged from research laboratories in the 1970s. Some of the challenges are logistical and legal, and have to do with management of domain names, intellectual-property agreements, and international business activities. Others are technical, resulting both because we are envisioning applications that the current Internet cannot support, and because the existing infrastructure cannot scale to a world in which a huge portion of the world's population is online and individual homes and businesses may have IP addresses for tens of electronic devices, such as appliances, heating systems, or security alarms.

In this presentation, I will discuss some of the US research and testbed activities that are currently underway in an effort to respond to the technical challenges. These include the Internet-2 testbed created by a consortium of academic institutions, and the federal government's Next Generation Internet research initiative. I will explain the difference between these two programs and identify some of the technical requirements other than a simple increase in bandwidth that have been identified for the evolving Internet.

This will lead to a discussion of the limitations of the current Internet that have constrained its use in health care and that accordingly help to define the networking research agenda that is of greatest importance to the biomedical community. Policy and regulatory issues that arise because of health care's use of the Internet will also be discussed, as will those technical requirements that may be unique to biomedical applications.

One goal of the discussion will be to motivate an international discussion of the ways in which the medical informatics community should be engaged in both basic and applied research in the area of networking and the future of the Internet."

